# Effect of bevacizumab on brain radiation necrosis in anaplastic lymphoma kinase‐positive lung cancer

**DOI:** 10.1002/rcr2.454

**Published:** 2019-06-24

**Authors:** Kengo Tanigawa, Keiko Mizuno, Yusuke Kamenohara, Taiji Unoki, Shunsuke Misono, Hiromasa Inoue

**Affiliations:** ^1^ Department of Pulmonary Medicine, Graduate School of Medical and Dental Sciences Kagoshima University Kagoshima Japan

**Keywords:** ALK, ALK‐TKI, bevacizumab, brain radiation necrosis, stereotactic irradiation

## Abstract

Central nervous system (CNS) metastases from anaplastic lymphoma kinase (ALK)‐positive lung cancer often results in failure of ALK‐tyrosine kinase inhibitor (TKI) therapy. Patients with uncontrolled CNS metastases receive radiation therapy, which sometimes causes brain radiation necrosis. We added bevacizumab (15 mg/kg, every 3–4 weeks) to the regimen of four ALK‐positive lung cancer patients with brain radiation necrosis who were receiving ALK‐TKI therapy. A decrease in brain radiation necrosis was seen in all the patients, and an improvement in symptoms was seen in three patients. In one patient who was receiving corticosteroid therapy, we could taper the dose and subsequently discontinue it. While one patient discontinued bevacizumab because of adverse events, the other three continued with the treatment. Therefore, the combination of bevacizumab with ALK‐TKI seems to be an effective, manageable, and tolerable treatment for brain radiation necrosis.

## Introduction

Lung cancer often results in brain metastases. In patients with advanced anaplastic lymphoma kinase (ALK)‐positive non‐small cell lung cancer (NSCLC) who did not receive systemic treatment, the incidence of central nervous system (CNS) metastases is 20–40%. 1, 2 CNS metastases are common in ALK‐positive NSCLC patients receiving ALK‐tyrosine kinase inhibitor (TKI) therapy, and brain metastases lead to failure of this treatment. Therefore, managing CNS metastases is important for a better prognosis.

ALK‐positive NSCLC patients with uncontrolled brain metastases are often treated with radiotherapy, which includes whole‐brain irradiation and stereotactic irradiation (STI). Although STI is administered to lung cancer patients with a few brain metastases, these patients go on to develop brain radiation necrosis. Corticosteroids, antiplatelet agents, anticoagulants, hyperbaric oxygen therapy, and surgery are used to treat brain radiation necrosis, but the efficacies of these treatments have not been proven. Recent reports have demonstrated the effectiveness of bevacizumab in treating brain radiation necrosis. 3, 4, 5 It improves cerebral oedema, helps reduce the corticosteroid dose, and provides symptomatic relief.

The combination of erlotinib, an epidermal growth factor receptor (EGFR)‐TKI, and bevacizumab has been shown to be effective and safe in patients with EGFR mutation‐positive NSCLC. 6 However, the safety and efficacy of the combination of ALK‐TKI and bevacizumab are unknown.

We assessed four ALK‐positive NSCLC patients who were treated with ALK‐TKIs. They developed brain radiation necrosis and brain oedema after STI. We report the efficacy and safety of bevacizumab given in combination with ALK‐TKIs for brain radiation necrosis in ALK‐positive NSCLC.

## Case Series

We examined four ALK‐positive NSCLC patients who received ALK‐TKI treatment and developed brain radiation necrosis from September 2016 through September 2017. They had brain metastases and were treated with the γ‐knife. After irradiation, they received ALK‐TKI, which resulted in the development of brain radiation necrosis. They underwent brain magnetic resonance imaging (MRI) and were diagnosed with brain radiation necrosis. We then administered bevacizumab in combination with ALK‐TKI to control brain radiation necrosis. Similar to the study of Seto et al. 6, we administered bevacizumab at a dose of 15 mg/kg every 3–4 weeks.

All the patients had a performance status of 1 and showed symptoms related to brain radiation necrosis. The histology of the four patients demonstrated adenocarcinoma. Two patients were positive for ALK based on both fluorescence in situ hybridization (FISH) and immunohistochemical staining, while the third and fourth patients tested ALK‐positive based on only FISH and immunohistochemistry, respectively. One patient received crizotinib as first‐line therapy, and three patients received alectinib as second‐line therapy. The number of brain metastases ranged from 2 to 11 (Table 1), and their diameters ranged from 20 to 45 mm.

**Table 1:** Characteristics and results of this case series.

	Case 1	Case 2	Case 3	Case 4
Age (years)	78	74	49	44
Gender	Male	Male	Female	Female
Performance status	1	1	1	1
Histology	Adenocarcinoma	Adenocarcinoma	Adenocarcinoma	Adenocarcinoma
Stage	Recurrence	Recurrence	IV	IV
Line	2	2	1	2
ALK‐TKI	Alectinib	Alectinib	Crizotinib	Alectinib
Symptom	+	+	+	+
Brain metastases (n)	6	2	4	11
Improvement in symptoms	−	+	+	+
Improvement in brain oedema	+	+	+	+
Improvement in brain metastases	+	+	+	+
Adverse events related to Bev	Hypertension, proteinuria	Hypertension	Oedema, hypertension, proteinuria	Proteinuria
Discontinuation of Bev	+	−	−	−
Duration from STI to BRN (days)	280	370	153	139
Duration from ALK‐TKI to BRN (days)	273	133	118	35
Duration from BRN to Bev (days)	281	141	55	142
Duration from Bev to radiographic response (days)	51	48	46	70

ALK, anaplastic lymphoma kinase; Bev, bevacizumab; BRN, brain radiation necrosis; STI, stereotactic irradiation; TKI, tyrosine kinase inhibitor.

**Figure 1:**
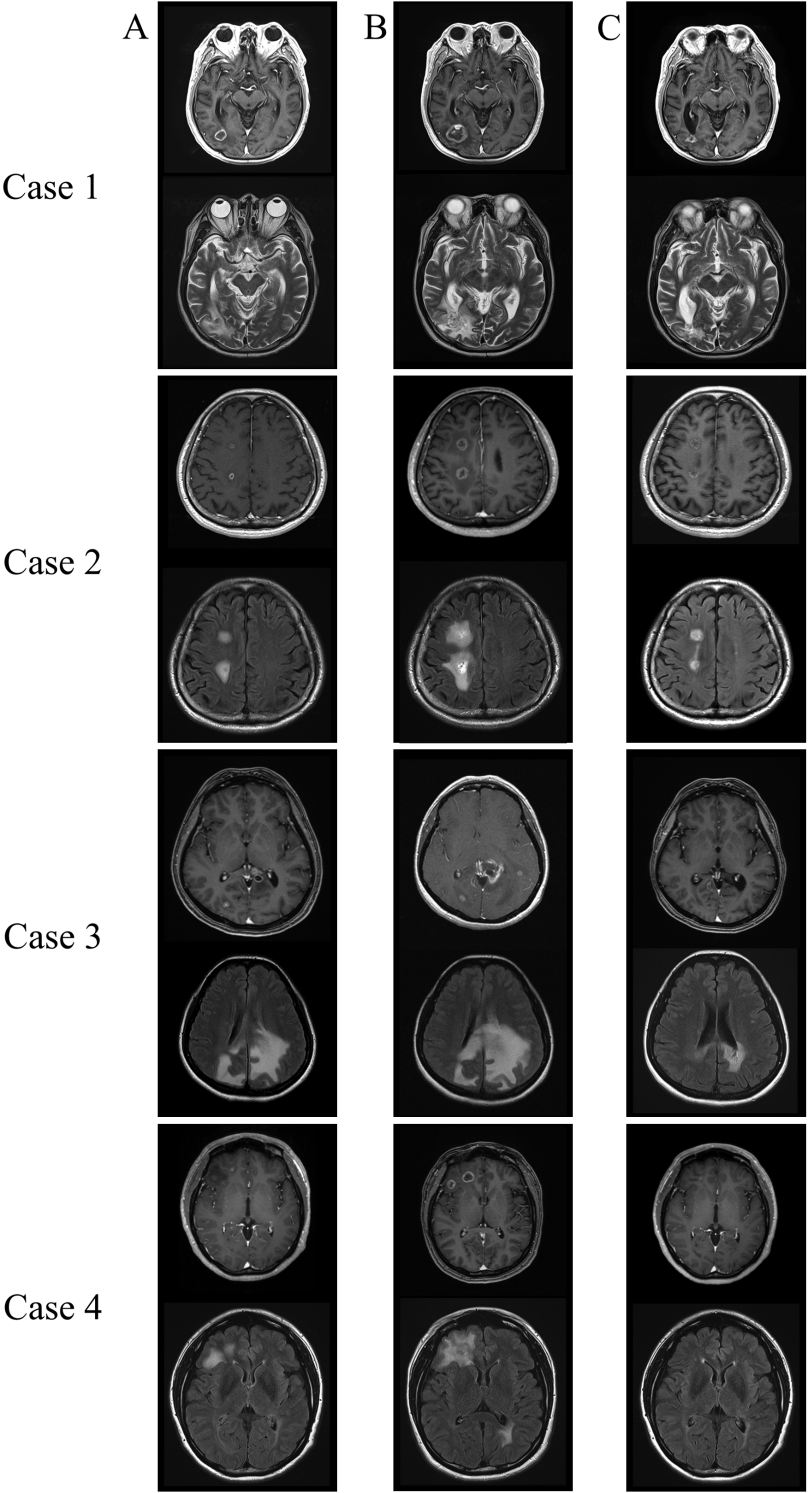
(A, B) Worsening of brain radiation necrosis and brain oedema post‐γ‐knife therapy administration of anaplastic lymphoma kinase‐tyrosine kinase inhibitor (ALK‐TKI). (B, C) Decrease in size of brain radiation necrosis and brain oedema after adding bevacizumab to the treatment regimen. Upper image shows T1‐weighted magnetic resonance imaging (MRI) after gadolinium injection, while the lower image shows T2‐weighted MRI for Case 1. For Cases 2–4, the upper image shows T1‐weighted MRI after gadolinium injection, while the lower image shows fluid‐attenuated inversion recovery (FLAIR) MRI.

The patients responded well to the treatment; three patients showed improvement in their symptoms. Brain oedema improved in all the patients, and brain metastases decreased in size (Fig. 1). Although only one patient received betamethasone, we tapered its dose gradually and discontinued it after adding bevacizumab to crizotinib treatment.

The adverse effects of bevacizumab included high blood pressure and proteinuria in three patients (Table 1). One patient who had grade 3 high blood pressure, grade 4 proteinuria, and lower leg oedema discontinued bevacizumab treatment, although his brain oedema had improved following a single dose of bevacizumab. The adverse events related to ALK‐TKI included rash, neutropenia, renal impairment, diarrhoea, visual deficit, nausea, and headache. However, no serious adverse events related to ALK‐TKI were observed in these cases.

The deterioration of brain oedema because of brain radiation necrosis occurred 139–370 days after the γ‐knife therapy. The interval between worsening of brain oedema and adding bevacizumab to ALK‐TKI ranged from 55 to 281 days. Brain oedema improved 46–70 days after adding bevacizumab to ALK‐TKI (Table 1).

## Discussion

The standard therapy for advanced ALK‐positive NSCLC patients is ALK‐TKIs. ALK‐TKIs are effective for intracranial metastases. In a previous report 2, CNS response was seen in 81 and 50% of the patients in the alectinib and crizotinib groups, respectively. The durations of intracranial response for alectinib and crizotinib were 17.3 and 5.5 months, respectively; CNS progression occurred in 12% and 45% of the patients treated with alectinib and crizotinib, respectively. In advanced ALK‐positive NSCLC patients, brain metastasis is the most common indicator of progressive disease 7 and was seen in 23.8, 45.5, and 58.4% of the patients at 1, 2, and 3 years 1, respectively.

When patients have CNS metastases, they are often treated with radiation therapy. Stereotactic radiosurgery (SRS), a part of STI, is often used to treat brain metastases. However, SRS leads to symptomatic brain radiation necrosis in 2–10% of treated patients 8, which is believed to be a result of damage to endothelial cells 9. Vascular endothelial growth factor (VEGF) induces angiogenesis and permeabilization of blood cells. 10 VEGF produced by the reactive astrocytes, localized mainly in the perinecrotic area, cause both angiogenesis and subsequent perilesional oedema typically found in brain radiation necrosis 11. Therefore, bevacizumab, an anti‐VEGF antibody, is effective against brain radiation necrosis and has shown clinical efficacy. 3, 4, 5 It has been suggested that an anti‐VEGF antibody changes tumour vascular function and tumour uptake of anticancer drugs and improves the delivery and efficacy of chemotherapy 12, 13. The effectiveness of ALK‐TKI improved by bevacizumab might decrease brain tumour size, which might alter brain radiation necrosis. In our patients, treatment with bevacizumab resulted in a decrease in brain oedema and improvement in symptoms. Previous reports showed that MRI responses were observed at 6–12 weeks 3, 4. Similar to the findings in the previous reports, the time to responses after bevacizumab therapy was 46–70 days in this case series. In one patient, we could reduce the dose of corticosteroids given. All the patients reported here received ALK‐TKI after the γ‐knife treatment, and all of them developed brain radiation necrosis. However, following the addition of bevacizumab to the treatment regimen, the brain metastases decreased in size, and brain oedema improved. Although managing brain metastases in ALK‐positive NSCLC patients is often difficult, combining STI and bevacizumab helped in controlling the brain radiation necrosis, allowing these patients to receive ALK‐TKI therapy for a longer time.

Bevacizumab administration was stopped in one patient because of adverse events. However, following a single dose of bevacizumab, the patient's brain radiation necrosis showed improvement, suggesting that a single bevacizumab dose can also be effective. In a phase 2 study, a combination of erlotinib plus bevacizumab resulted in grade 3 or higher adverse events, the most frequent ones being hypertension and proteinuria 14. Consistent with this previous report, hypertension and proteinuria were the most frequently reported adverse events among our four patients as well. In two previous clinical trials of bevacizumab for brain radiation necrosis 3, 4, 5, the patients received bevacizumab at 5 mg/kg every 2 weeks, 7.5 mg/kg every 3 weeks, or 7.5 mg/kg every 2 weeks; however, we administered it at 15 mg/kg every 3–4 weeks. The high dose of bevacizumab used in our study could have caused severe adverse events, therefore leading to the need to discontinue it in one patient. However, in another patient, the bevacizumab dose was reduced due to adverse events, which allowed her to continue with the treatment. Therefore, if we had used a lower dose of bevacizumab, treatment continuation could have been possible in the patient who discontinued it. The other three patients, who had hypertension and proteinuria, could tolerate the adverse events and continued receiving bevacizumab, which suggests that treatment with ALK‐TKI and bevacizumab is tolerable and effective.

This case series has some limitations. First, it evaluated a small number of patients from a single facility. Second, it was difficult to distinguish brain radiation necrosis from worsening of brain metastases. In this case series, the patients only underwent MRI examinations; we did not perform positron emission tomography or single‐photon emission computed tomography. The patients might have shown worsening of brain metastases. Third, because of the adverse events related to ALK‐TKI, the ALK‐TKI dose was reduced in two patients, which might have worsened the brain oedema and brain metastases. Dose, intervals, and duration of administration of bevacizumab were different in each clinical trial and were not determined. In this case series, one patient discontinued bevacizumab because of adverse events. Therefore, dose, intervals, and duration of bevacizumab may be important for maintaining their effectiveness and managing the adverse events. Although combining bevacizumab with ALK‐TKI resulted in a radiographic response, improvement in symptoms, and reduction in corticosteroids dose, its effects on the prognosis were not confirmed. Clinical studies with larger patient populations are needed to confirm these findings.

### Disclosure Statement

This study was approved by the Institutional Review Board.
